# Adolescent Development and the Parent–Adolescent Relationship in Diverse Family Forms Created by Assisted Reproduction

**DOI:** 10.3390/ijerph192416758

**Published:** 2022-12-14

**Authors:** Maria Quintigliano, Nicola Carone, Anna Maria Speranza, Annalisa Tanzilli, Roberto Baiocco, Lavinia Barone, Concetta Pastorelli, Vittorio Lingiardi

**Affiliations:** 1 Department of Dynamic and Clinical Psychology, and Health Studies, Sapienza University of Rome, Via degli Apuli 1, 00185 Rome, Italy; 2 Department of Brain and Behavioral Sciences, University of Pavia, Piazza Botta 11, 27100 Pavia, Italy; 3 Department of Developmental and Social Psychology, Sapienza University of Rome, Via dei Marsi 78, 00185 Rome, Italy; 4 Department of Psychology, Sapienza University of Rome, Via dei Marsi 78, 00185 Rome, Italy

**Keywords:** assisted reproduction, parent–child relationship, adolescent development, lesbian mothers, gay fathers, single parents, identity formation, heterosexual parents, attachment

## Abstract

Assisted reproduction techniques (ARTs) are employed by single individuals and couples who are not otherwise able to conceive spontaneously. While the use of ARTs is increasing, research is lacking on the attempts made by adolescent offspring conceived via ARTs to integrate their ART conception into their identity and negotiate a connection with, and autonomy from, their parents. The present article reviews studies investigating adolescent development and the parent–adolescent relationship in diverse family forms created by ARTs (mainly heterosexual and lesbian parent families), and discusses the results in light of attachment, identity development, and emotional distance regulation theories. Overall, the results indicate that the psychological adjustment of adolescents conceived via ARTs is not undermined by the manner of their conception, and that they enjoy positive relationships with their parents with no difference from those enjoyed by spontaneously conceived adolescents. However, it remains unknown whether the development of a reproductive identity in adolescence is likely to influence adolescents’ interest in searching for or contacting their donors, surrogates, and/or donor siblings. The results suggest the relevance of considering the parent–adolescent relationship, disclosure, and identity formation issues when planning psychological counseling and support interventions with ART parents and their adolescent offspring, and emphasize the need to further investigate these aspects in diverse ART families, including single-, gay-, bisexual-, and trans*-parent families.

## 1. Introduction

An increasing number of single individuals and couples of diverse genders, gender identities, and sexual orientations (e.g., heterosexual couples, single men and women, trans* individuals, lesbian women, and gay men) are having children through assisted reproduction techniques (ARTs (Note that the acronym may vary across countries worldwide. Here we used the US acronym of Assisted Reproduction Techniques)) [1]. ARTs include both homologous and donor-dependent reproductive techniques. *Homologous reproductive techniques* (i.e., involving the egg and sperm of the intended parents) include *in vitro fertilization* (IVF), wherein the egg is fertilized in vitro; and *intracytoplasmic sperm injection* (ICSI), wherein the sperm is injected into an egg. ARTs involving donated gametes include *donor insemination* (i.e., the male gametes are donated), *egg donation* (i.e., an egg donor is used), and *embryo donation* (i.e., both sperm and egg are donated). Finally, *surrogacy* describes an arrangement in which a woman carries the pregnancy with the intention of handing over the resulting child to the intended parent(s).

It is estimated that, worldwide, more than 9 million infants have been born as a result of ART procedures since 1978 [2]. In parallel, the last two decades have seen the establishment of equal marriage legislation in 30 countries [3]. Such legislation has provided sexual-minority couples with important legal protection [4] and symbolic recognition of the validity of their families, and in some cases has opened previously unavailable routes to parenthood.

Although ART conception can bring great joy to individuals who may not otherwise be able to achieve parenthood, many concerns have been raised about certain characteristics of the intended parents (e.g., older age, unpartnered, nonheterosexual orientation) and the practical consequences of ART techniques (e.g., reproductive tourism) [5]. Further concerns have been raised that parents (particularly heterosexual-parent families formed via gamete donation) may have no intention of disclosing (or may postpone disclosure of) the assisted conception to their child [6]. Additionally, some perspectives caution that a child’s lack of a genetic relationship with their mother (in the case of egg donation) or father (in the case of donor insemination), or both parents (in the case of embryo donation) may be problematic (for a discussion, see Golombok, 2020) [1].

From a psychological perspective, it remains that, regardless of the intended parent(s)’ characteristics, the most salient aspect of the use of ARTs is that it often requires parents to face hard decisions and emotionally charged experiences, which may later be reflected in the adjustment of their child. In this regard, the European Society for Human Reproduction and Embryology (ESHRE) has recommended mandatory assessment and counseling for intended parent(s), focusing “on the best interest of the child” [7].

The past few decades have seen a growing research interest in the medical outcomes of children born through ARTs [8]. However, relatively few studies have examined the psychological effects of ART conception on parents and their children, and most of these have focused on infancy and childhood (for an exception, see Ilioi and Golombok, 2015 [9]). Overall, these studies have shown that families formed via ARTs show good family functioning when children are in infancy and childhood, with well-adjusted and competent parents and healthy children [1].

In a similar vein, the extensive literature on lesbian-mother families created by donor insemination and the smaller (but growing) literature on gay-father families created by surrogacy with preschool- and school-age children suggests that parenting quality and child adjustment are unrelated to parents’ sexual orientation in these developmental periods [10,11]. Rather, parenting quality and child adjustment relate to family processes (e.g., family communication, the couple relationship) and external events (e.g., stigmatization) [12,13]. In some cases, preschool and school-age children of sexual-minority parents have been found to demonstrate better psychological adjustment than their peers in heterosexual-parent families [14,15,16].

There has been little focus on parenting and child development in single- and trans*-parent families created by ARTs, though the preliminary evidence on children in early and middle childhood indicates that neither the number of parents nor parents’ gender identities result in negative outcomes [17,18,19,20,21]. However, some studies have shown that a single-father family structure created by surrogacy and parental gender identity are, in some cases, relevant to parents’ and children’s external experiences, including their exposure to microaggressions and negative attitudes from others [22,23].

It remains unknown whether all of these findings pertaining to families of diverse parent number, gender, gender identity, and sexual orientation are maintained as children enter adolescence. Evidence to this effect would be particularly relevant for determining whether some aspects of parenting quality and child adjustment might differ across children’s developmental stages and/or family types. In light of the aforementioned knowledge gaps, the current article offers an overview of adolescent development and the parent–child relationship in diverse family forms created by ARTs, according to current empirical research. To this end, the attachment, identity development, and emotional distance regulation frameworks are introduced to provide a theoretical explanation of key processes involved in ART families during adolescence.

## 2. Theoretical Frameworks for Understanding the Development of Adolescent Offspring Born through Assisted Reproduction

The developmental period of adolescence is a useful focus for studies on the effects of ART conception on adolescent adjustment and the parent–child relationship, as it is precisely in this sensitive period that adolescents face important developmental tasks regarding identity formation and the negotiation between connection with, and autonomy from, parents, also related to the development of other important capacities such as mentalizing, emotional regulation, and peer relationships [24]. Adolescents’ success or failure in achieving these tasks can result in varying degrees of conflict, with profound implications for adult development [25,26]. In particular, adolescents’ greater desire for autonomy may present difficulties in families formed via ARTs, as parents in these families tend to be very involved with their children [27].

Similarly to adopted children, donor-conceived adolescents who are aware of their manner of conception may feel challenged in their efforts to understand themselves as having a genetic connection to a donor and possibly donor siblings (i.e., genetic half-siblings born from the same donor but raised in different families), whose identities they may never know. Adolescents born through surrogacy face similar challenges in relation to their gestational connection to the surrogate (and their genetic connection to her, if they were conceived using the surrogate’s egg) or their genetic connection to an egg donor, as well as their gestational or genetic link to the surrogate’s own children.

Attachment theory [28] holds that the quality of the child–parent relationship is crucial for the adolescent, insofar as a secure attachment pattern can contribute to a healthy transition to autonomy [29,30]. Moreover, in adolescence (as in early and middle childhood), a secure attachment pattern is associated with secure exploration [31] and is likely to protect against many risky behaviors and psychopathology [32,33,34,35,36,37]. Overall, these considerations recall the *secure base phenomenon* [38], which is a key tenet of attachment theory, defining the purposeful balance between children’s use of their parents as both a secure base from which to explore and learn about their surroundings and a safe haven to return to if a threat arises or fatigue or illness hits. Similarly, Grossmann et al. [39] introduced the companion idea of secure exploration to refer to “a child’s ability to organize emotions and behaviors open-mindedly, non-defensively, and with concentration when responding to ‘curious’ events, and to do so with care; and the child’s confidence in an attachment availability and helpfulness, should help be needed” (p. 859).

From this perspective, insecure adolescents born through ARTs may perceive any exploration of their origins (e.g., reflecting on their thoughts and feelings towards the donor and/or the surrogate, initiating conversations about their genetic origins and/or family structure) as threatening and intimidating because such exploration is new and unfamiliar, and because they do not know how their parents might react to their curiosity. Conversely, secure adolescents may feel more comfortable expressing their desire to know more about their origins, and be less likely to feel that this curiosity will risk their emotional bond with their parents [40,41,42].

Additionally, during adolescence, the physical maturation of puberty marks an important and irreversible change in the adolescent’s life: at this point, the adolescent is biologically able to become a parent in their own right. Moreover, the hormonal changes brought on by puberty affect mental functioning and generate more active sexual interest, bringing focus to issues of reproduction [43]. It remains unknown whether the development of a reproductive identity in adolescence is likely to influence adolescents’ interest in their donors and/or surrogates, as shown by research with adopted adolescents and their biological parents [44]. Finally, in adolescence, an understanding of who one is in relation to others becomes key for the formation of identity, as theorized by Erikson [25]. In this respect, one might wonder if adolescents’ self-definition processes could be affected by their understanding of their origins, which may include adoption or assisted conception [9,45], or (with regard to adolescents conceived via ARTs) adolescents’ contact with and knowledge about their donors, surrogates, and/or potential donor siblings.

Grotevant’s conceptualization [46] of *emotional distance regulation* in adoptive families may provide insight into the ways in which parents and their adolescent offspring born through ARTs might manage and negotiate closeness and distance with each other and with donor(s), a surrogate, and donor siblings. In fact, the relationships between (intended) parents, children, egg donors, sperm donors, surrogates, and donor siblings are multifaceted and often emotionally involved [47,48]. Donors may be motivated by a desire to help single individuals or couples have children, with the view that a family is, above all else, a relational system defined by care rather than genetics. Some studies have found that donors consistently report altruism and the wish to help another couple have a child to be significant motivating factors for donating [49,50,51,52]. In other studies on donors’ and surrogates’ motivations, altruism and empathy for intended parents may coexist with financial compensation [49,52,53]. Moreover, in the specific case of egg-share donation, donating for the purpose of obtaining cheaper treatments for themselves is equally important [54], with some egg-share donors feeling this process to be a “win win” for all parties and considering it preferable for eggs to come from women already undergoing IVF [49]. Some donors may maintain a certain physical and emotional distance from the family they helped to create [54], considering the act of donating similar to that of giving away a cell. However, embryo donors may prove an exception to this, as they are more likely to consider their embryos potential children [55].

At the same time, (intended) parents and adolescents need to define a comfortable proximity to the donor, the surrogate, and/or any donor siblings: for example, they may try to create a sense of familiarity by attributing to these parties their own personal values or other shared interests and characteristics [56,57,58]. It is of fundamental importance that offspring’s views on their relationships with donors, surrogates, and donor siblings be explored [23,59]. This may be particularly relevant for adolescent offspring, whose identity formation processes are likely to rely on their definition of their origins, and who may be at an age at which it is legal (i.e., 16 or 18 years, depending on the jurisdiction) to access a donor’s identity.

## 3. Adjustment of Adolescents Born through Assisted Reproduction

A number of studies have focused on the physical health, cognitive development, and socioemotional competencies of children conceived through ARTs. Two literature reviews [60,61] considering some important confounders (e.g., multiple births, prematurity) have found that IVF children do not differ from spontaneously conceived children in physical health. However, further and longitudinal research is needed to confirm these findings.

With regard to neuropsychological and cognitive development, no differences have been found in psychomotor abilities, social competencies, language progress, and behavioral development between children born through ARTs and spontaneously conceived children [62]. Moreover, a recent review underlined the importance of considering single versus multiple births in the evaluation of cognitive development in children born through ARTs [62]. Although some studies have found that ART conception has some negative influence on cognitive development (e.g., lower intellectual quotient) [63], the differences between children conceived via ARTs and spontaneously conceived controls have been found to disappear when only single children are considered [64,65]. For instance, the association between attention deficit hyperactivity disorder (ADHD) and IVF conception has been shown to disappear when only single children are analyzed [66]. Additionally, no higher risks for being on the autism spectrum or emotional and behavioral disorders have been found in children born through ARTs compared to spontaneously conceived children [64,67].

In relation to behavioral and psychological outcomes in middle childhood, a recent study observed no differences between children born to gay or heterosexual single fathers through surrogacy, gay partnered fathers through surrogacy, and heterosexual partnered fathers through IVF [18]. Furthermore, a systematic review of psychological adjustment among adolescents conceived through ARTs and raised in heterosexual- or lesbian-parent families summarized the evidence according to whether the parents used their own gametes or reproductive donation [9]. The results indicated that, regardless of their family type, adolescents born through ARTs seemed equally well-adjusted, relative to spontaneously conceived adolescents and standardized normative samples.

Following the same distinction criterion (i.e., use of homologous vs. donation-based ARTs), previous research has reported no differences between IVF adolescents and spontaneously conceived controls in terms of cognitive ability and school performance [68,69]. However, a recent systematic review [68] found that studies on ICSI children have not been consistent, and that most previous studies have suffered from important methodological limitations and been too heterogeneous in their assessment of cognitive outcomes; thus, there is a limit to the generalizations that can be drawn. Other studies have found that IVF adolescents do not display differences in behavioral problems [70], peer problems [71], and emotional functioning [72], even when considering both single and twin IVF adolescents [73].

With regard to reproductive-donation families, a nationwide registry-based cohort study in Denmark found that adolescent offspring showed good academic achievement [74,75]. Similarly, a UK study by Golombok et al. [76] found that adolescents conceived via ARTs showed healthy psychological adjustment. More specifically, the UK study found that, when offspring were aged 14 years, mothers in surrogacy families showed less negative parenting and reported greater acceptance of their adolescent children and fewer problems in family relationships, compared with gamete-donation mothers [76]. Additionally, less positive relationships were found between mothers and adolescents in egg-donation families than in donor-insemination families. However, there were no differences in adolescent adjustment problems, psychological well-being, and self-esteem between donor-insemination, egg-donation, surrogacy, and spontaneous-conception families.

In a recent study conducted in Western Australia by Wijs et al. [77], ART-conceived offspring reported fewer externalizing problems at ages 14 and 17 relative to their spontaneously conceived counterparts. Additionally, at both ages, no differences in internalizing behavior emerged from adolescent or clinical reports, with their scores being below the clinical cut-off; however, parents of ART-conceived adolescents reported more internalizing problems in offspring. The higher percentage of ART-conceived adolescents with clinical depression at age 14 was no longer apparent at age 17. An explanation for these findings may stem from the tendency for parents who conceive through ARTs to be overprotective, see their children as precious, and have higher expectations of their children, which in turn can affect their children’s behavior [72]. Two further underlying mechanisms for these differences in health outcomes may be the higher prevalence of obstetric complications in ART pregnancies [78] and the epigenetic alterations that occur around the susceptible window of conception during the ART process [79,80,81]. In more detail, the epigenetic changes that alter the fetal programming of endocrine and metabolic processes may lead to altered activity of the hypothalamic–pituitary–adrenal axis, which is involved in the regulation of stress and arousal, and thereby the regulation of emotion and behavior [72].

Another relevant theme for ART families and adolescent adjustment is the disclosure of assisted conception [6]. While sexual-minority and single parents are unlikely to conceal their use of reproductive donation due to the presence of same-gender parents or the absence of a second parent, respectively, heterosexual couples may find it easier to do so. Previous studies in the UK have not found negative effects of secrecy on donor-conceived adolescents’ psychological adjustment [82,83]. However, a more recent UK study [84] underlined that children who are told about their origins at a young age are better psychologically adjusted and report higher family relationship quality in adolescence.

The few studies on sexual-minority parents with adolescent offspring have been mainly conducted by the National Longitudinal Lesbian Family Study (NLLFS) group [85], showing that the psychological adjustment of the adolescent offspring of lesbian mothers seems unaffected by the disclosure of their conception method [12,86,87,88]. In fact, one study comparing the adolescent offspring of lesbian mothers conceived through donor insemination and the adolescent offspring of heterosexual parents conceived through spontaneous conception in the Netherlands found the former to demonstrate higher levels of self-esteem and fewer conduct problems relative to the latter [89]. In a similar vein, the only study on children born to single fathers through surrogacy was conducted in Italy when children were aged 6–12 years [18]; the findings showed that it was not disclosure, per se, that was associated with children’s behavioral adjustment, but children’s weaker understanding of surrogacy, lower satisfaction with their contact with the gestational carrier, and lower comfort with their family arrangement. It is unknown whether these findings might also apply to gay-father families created by surrogacy.

To the best of our knowledge, very little research has been conducted on possible protective or risk factors in the disclosure process in families formed by ARTs. From an attachment perspective [28], ART-conceived children and adolescents with secure attachment might be expected to manage the disclosure in a less distressing way, due to the interiorization of their parents’ availability in case of need and vulnerability. Attachment security would also imply that these offspring may feel more comfortable and less constrained in asking their parents questions about the donor or surrogate, because they have internalized their parents as a secure base from which to explore their ART origins, without risking the emotional bond with their parents [40,42,90]. Thereby, insecurely attached children or adolescents may have more difficulty processing their ART conception following disclosure. On the one hand, offspring with an *avoidant attachment pattern* may be more likely to perceive mistrust in relation to others and react to disclosure with a hypoactivation of the attachment system, feeding an idea of others as unreliable and of the self as not worthy. On the other hand, offspring with a *preoccupied attachment pattern* may be curious about their donor or surrogate, but feel guilty exploring this due to entangled or role-reversed relationships with their parents.

Preliminary confirmation of these hypotheses derives from three studies of lesbian and single mothers with children conceived through donor insemination in the USA and the UK [42,90], respectively and gay fathers with children born through surrogacy in Italy [40]. Overall, these studies have indicated that in middle childhood [40] and adolescence [42,90], offspring’s attachment security is associated with a greater interest in exploring their ART conception. In addition, adolescent offspring with higher levels of disorganized attachment to their single and lesbian mothers are more likely to perceive their sperm donor—and donor conception in general—negatively [42,90], and offspring of lesbian mothers with a dismissive attachment pattern are less likely to express curiosity [42]. These findings suggest that attachment theory may be able to provide unique explanations for peculiar family dynamics in ART families. However, further research from this perspective is needed.

## 4. The Parent—Adolescent Relationship in Families Formed through Assisted Reproduction

No prior research on parent–adolescent relationship quality has been conducted with ICSI families. However, some studies have examined this factor among IVF families created using homologous reproduction. In their systematic review, Ilioi and Golombok [9] reported that most studies have not found differences in the parent–adolescent relationship between IVF and spontaneously conceived families, particularly with respect to warmth and conflict [71,91]. While a more indulgent education style and lower maternal sensitivity have been reported for IVF families [91], these seem to be related to the experience of infertility, and not to the type of ART used [92]. Furthermore, some differences have been described according to the type of ART used, with parent and child gender, child age, and disclosure representing key mediators of the parent–adolescent relationship in families created via donor insemination [9].

While a positive relationship has been found between lesbian mothers and their adolescent offspring conceived through donor insemination [93], most studies on the parent–adolescent relationship have been conducted with heterosexual-parent families created by reproductive donation. In relation to these families, research has examined whether the child’s lack of genetic relation to a parent may determine a change in the parent’s feelings or behavior towards that child, and whether this, in turn, may influence the child’s adjustment and identity formation in adolescence (for a discussion, see [9]). In particular, fathers of children conceived through donor insemination have been shown to keep a greater distance from their children compared to fathers of children conceived through spontaneous conception [94,95].

The issue of disclosure raises further concerns, as many heterosexual ART parents feel uncertain about whether and when to disclose the lack of a biological link with their ART-conceived children [6]. Discomfort over the secrecy surrounding heterosexual-parent families created using ARTs has been the topic of clinical and research attention, due to its potential interference with family relationship quality and child development [94,96]. Regarding this, a UK study of donor-insemination families found no difficulties in the mother–adolescent relationship between IVF, adoptive, and spontaneous-conception families [91]. Of note, although the two offspring in the study who had been told of their donor conception in middle childhood found the disclosure upsetting at the time, they no longer experienced distress at 18 years of age.

In general, the literature indicates that the age at which children are told or discover that they were born through ART influences their feelings about the circumstances of their birth, with those who are told later (i.e., from adolescence onwards) or who discover accidentally experiencing a greater likelihood of psychological distress [59,97]. Currently, disclosure about donor conception is recommended, since research has found lower levels of conflict between mothers and adolescent sons who are aware of their origins, even compared to mothers and adolescent daughters [27].

The empirical evidence on family functioning in heterosexual-parent families created by egg donation comes almost exclusively from two longitudinal studies of children conceived via anonymous egg donation and assessed at the ages of 3–8 and 12 years in the first study [83,98], and 1, 2, 3, 7, 10, and 14 years in the second study (for a review, see [1]). Both studies found egg-donation families to be functioning well in terms of parenting quality and child adjustment. A more recent UK study of families who conceived using identity-release egg donation found a lower quality of mother–infant relationship within egg-donation families compared to IVF families; however, these differences disappeared when twins were excluded from the sample [99].

With regard to adolescents conceived through egg donation, one study found that egg-donation mothers were as sensitive to their adolescents’ needs as IVF mothers [83], though less emotionally involved compared to donor-insemination mothers. In contrast, another study found that egg-donation mothers and their adolescent offspring reported a less positive relationship quality compared to donor-insemination mothers and their adolescent offspring, though this difference did not bear out in the observational assessment [76]. Furthermore, age of disclosure about the method of conception has been shown to play an important role in child adjustment in families created by reproductive donation [84]. It is noteworthy that since the infertility stigma has been found to affect women more than men [100], this aspect may be relevant to explore with mothers of children conceived through egg donation in future research.

In relation to the use of surrogacy in heterosexual-parent families, a few studies have focused on the parent–adolescent relationship, finding a more positive mother–adolescent relationship in surrogacy families compared to gamete-donation families when children are aged 14 years. More specifically, mothers in surrogacy families have been found to show lower levels of negative parenting and greater acceptance towards their adolescents, and surrogacy families have been found to have better overall relationship functioning [76]. A longitudinal study by Golombok et al. [101] showed that surrogacy had no detrimental effect on parenting quality during childhood; rather, higher levels of maternal and paternal adaptation to parenthood were found [102]. To the best of our knowledge, no study has focused on the parent–adolescent relationship in gay-father families through surrogacy.

## 5. Extended Family Networks in ART Families with Adolescent Offspring

In the context of surrogacy and egg, sperm, and embryo donation, another emerging topic of interest relates to extended family networks, which may include donors, surrogates, and their families, as well as donor/surrogate siblings. The term *donor/surrogate sibling* is controversial, because not all people conceived through reproductive donation consider other people born via the same donor or surrogate to be siblings. Nevertheless, an increasing number of people born via egg or sperm donation are interested in searching for and contacting other people born via the same donor, to better understand themselves and their genetic identity [59]. There is no current evidence on whether offspring who share the same surrogate are interested in contacting and/or meeting one other.

It is of note that the spread of online donor registries is helping donor offspring search for, and connect with, donor siblings [103]. Some studies have found that ART offspring consider contact with donor siblings to be either normal or a unique experience, and adolescent ART offspring in particular associate it with identity formation [57]. However, some adolescents refer to the relationship with their donor siblings as turbulent, especially at the outset [57].

Considering Grotevant’s [46] conceptualization of *emotional regulation distance*, it may be possible to understand the attempts of some ART adolescents to contact their donor, surrogate, and/or donor siblings as an emotional regulation strategy, expressed through a search for closeness. Some ART adolescents, for example, may find similarities with people who are genetically linked to them reassuring, contributing to a more defined and integrated identity. However, other ART adolescents may fantasize about their donor or surrogate as a further parental figure when they have issues or conflict with their parents or when they need to (defensively) negotiate dependence on their parents (such as in adolescence, when individuals typically seek greater autonomy) [41]. Some adolescent and parent characteristics (e.g., state of mind with respect to attachment, personality traits) might be important moderators of how offspring search for and contact their donors, surrogates, and donor siblings. Future research should seek to explore this in more depth.

It remains unpredictable whether ART adolescents will be interested in searching for or contacting their donor siblings, and, if so, whether they will consider them actual siblings [104]. This is because individuals may have widely differing opinions on the importance of a genetic or gestational link within relationships. Furthermore, adolescents may hold different views from their parents, even about the search for donors, as indicated by the fact that not all donor-conceived offspring tell their parents about their interest in knowing/contacting their donor [59]. Appropriate terminology for donor-mediated relationships seems to be lacking, as not all donor-conceived offspring recognize themselves in the current language. Further research is needed in this regard.

## 6. Future Directions and Clinical Implications

Driven by the public debate over whether child adjustment and parent–child relationship quality are at risk in ART families, most studies have adopted a between-difference approach, considering “traditional” heterosexual-parent families with spontaneously conceived children the baseline against which to compare, interpret, and understand ART families. While the results have been reassuring with respect to concerns raised against ART families, research has been slow to move beyond a comparative lens, and thus the nuanced family dynamics and unique strengths of ART families have been overlooked. Future research should adopt a within-difference approach, applying family, clinical, and developmental psychology frameworks to empirically inform practitioners about how they might best support ART parents, children, and the entire family system over the life cycle.

From a theoretical perspective, the studies discussed in this article provide insight into the influence of family structure on child development and parenting, showing that the most critical factors for adolescent and parental flourishing are family processes (e.g., decision making related to the disclosure of ART conception) and extrafamily processes (e.g., infertility-related stigma, legal regulations). Taken together, the research findings align with previous evidence that the number, gender, gender identity, and sexual orientation of parents, and biological (un)relatedness between a parent and child are not influential per se [1,11,12].

That said, the structural distinctions between family types applied in the present article are imperfect, because the number, gender, gender identity, and sexual orientation of parents and parent–child biological (un)relatedness are not mutually exclusive in defining families, but in some cases conflate. However, these categories best reflect the distinctions used in previous research, e.g., [9]. In this vein, ART conception may be considered a specific modality through which individuals’ access to parenthood, and aspects and consequences of their identity and family constitution are not easily distinguishable from those linked to the lack of a genetic link with a child or the impossibility of conceiving. Under these circumstances, parent number, gender, gender identity, and sexual orientation may describe unique variations in family processes, motivating further research on diverse family forms created by ARTs.

It is worthy of note that the investigated studies were mainly limited to adolescents born to heterosexual parents through diverse ARTs and adolescents born to lesbian mothers through donor insemination. However, because some middle-childhood processes can lay the foundation for significant tasks in adolescence (e.g., achieving greater autonomy from parents), some studies on ART families, gay-father families with children born through surrogacy, and single-father families with school-age children born through surrogacy, were also included.

Given that advancements in reproductive medicine, more inclusive legal regulations, and societal acceptance have only occurred recently in some countries, no sufficient data exist on nonheterosexual-parent families with adolescents, and especially on gay-, single-, bisexual-, and trans*-/nonbinary-parent families after gender transition. Additionally, because of the increasing use of reciprocal IVF (i.e., whereby an embryo created using one parent’s egg is gestated by the other parent) in lesbian couples [93], it will soon be possible to examine adolescent adjustment and the parent–adolescent relationship in this emerging family type. It remains unknown whether technological advancements such as artificial gametes, mitochondrial replacement therapy, and social egg freezing will significantly affect parenting and child development.

From a methodological perspective, the increasing use of multimethod (i.e., in-depth interviews, observational assessments of parent–child interactions, standardized questionnaires) and multi-informant (i.e., data collected from the perspectives of both parents and adolescents) approaches may enable a multifaceted and more precise assessment of adolescent adjustment and parent–adolescent relationship quality. In particular, data collection from both parents in each family is aligned with the call for family researchers to consider the network of relationships in which the child is immersed [105]. Such an approach may facilitate an examination of the specific role played by each parent in the family system, and whether this caregiving role uniquely influences child development and parent–child relationship quality. Importantly, future research should overcome the tendency to operationalize the family as a unit of parents and the target child, and also consider the role of siblings, grandparents, and other family members.

Because of the time- and money-consuming nature of multimethod and multi-informant approaches, most of the examined studies involved a small sample size, which affected the power achieved by the statistical analyses. With few exceptions, e.g., [74,75], the studies could not draw data from nationally representative datasets, because method of conception is considered a private family matter. Additionally, in some countries (e.g., Italy), parents’ sexual orientation and gender identity are considered sensitive information, and are not collected at a national level. As a result, many of the studies relied on convenience samples and were likely susceptible to sampling bias, with families who were better functioning or who perceived less stigma around their family potentially more likely to participate.

Future studies would benefit from the use of qualitative methods to gather richer and more holistic insight into parents’ and children’s perspectives on how specific elements of their family structure may or may not influence family processes. That is, whether the number, gender, gender identity, or sexual orientation of parents or parent–child biological (un)relatedness are moderators (rather than predictors) of the adolescent–parent relationship and family outcomes. Furthermore, because donor-conceived adolescents are close to the age at which they may be legally authorized to access the identity of their donor (in the case of identity-release gamete donation), future qualitative research with ART families should also consider the impacts of the law on these families, gathering views from parents and adolescents themselves.

Similarly, in jurisdictions where surrogacy is banned, intended parents who are pursuing this path to parenthood must do so overseas. The experiences and perspectives of children born through cross-border surrogacy, whose surrogate and egg donor are from a different country, culture, and ethnicity, and may even speak a different language, require further research in relation to the modalities and level of contact, and satisfaction with the contact. Finally, parents’ discussions with their children about their family structure and ART conception, and the long-term effects of such socialization on their children’s aspirations and future plans of parenthood from adolescence onwards, as their reproductive identity develops and consolidates, warrants investigation.

The examined research was carried out using primarily Western and Westernized samples, which limits the extent to which the findings can be generalized to families in other geographic regions [106,107]. In light of the rapid growth in international fertility procedures worldwide [108], it is important that future research include families from more diverse geographic locations. Similarly, in terms of sociodemographic variables, the participating families were primarily white, financially stable, and highly educated. Although this profile is representative of most ART families, and particularly those who pursue cross-border services [109], the recruitment of more sociodemographically diverse samples would allow for a deeper examination of the effect of race and socioeconomic status factors—and their interaction with parent number, gender, gender identity, and sexual orientation, as well as biological (un)relatedness with the child—on child, parent, and family outcomes [11,110].

To summarize, some areas identified in this review still need further research: in particular, we refer, first, to the use of attachment theory as a framework to explain the peculiar family dynamics in ART families; second, to the need to adopt appropriate terminology for donor-mediated relationships coherently with the need of donor-conceived offspring to be recognized with specific language; third, to consider parent number, gender, gender identity, and sexual orientation as variation factors in family processes; fourth, to deepen understanding of how modalities and levels of contact may influence satisfaction in surrogacy.

Some reflections that may have clinical implications for work with ART families are worthy of note. Specifically, parents of children born through ARTs may have very different experiences regarding the difficulty of conceiving spontaneously. In some cases, they may show a strong desire to have a child and deep reflection around their decision to take this path; in other cases, feelings of impotence related to the condition of infertility may undermine the parent–child relationship. Therefore, research should aim at differentiating between these conditions, to generate greater insight into which factors are more influential for the quality of the parent–child relationship.

In a similar vein, the relationship with the donor, the surrogate, and/or donor siblings may be viewed through the modality of interpersonal emotional regulation [46] to inform psychotherapeutic work with ART offspring. As ART children age and become sufficiently mature to handle the relationships with their donors, their surrogates, and donor siblings independently of their parents, research should address the potential implications of this independent relationship management for the parent–child relationship. It also cannot be ignored that, as in all family types, in ART families, parents’ personality and psychological functioning, as well as particular aspects of family processes (e.g., family communication, family cohesion), can have a profound impact on the adjustment of offspring and, particularly in ART families, the acquisition of an autonomous identity during adolescence [111,112]. None of these aspects has been investigated, despite being potentially decisive for psychological counseling and support interventions with parents of children born through ARTs. As a final note, it could be informative to explore whether—and to what extent—the difficulties encountered by parents of children born through ARTs in procreating and their emotional experiences of infertility (particularly regarding heterosexual parents), as well as the barriers to accessing ARTs domestically and across borders (particularly by sexual-minority parents, gender-minority parents, and single parents) impact offspring’s sense of a reproductive self during the developmental stage of adolescence, which is decisive for one of the subsequent stages, which is centered on generativity [25].

## 7. Conclusions

The research described in the previous sections has consistently shown that the psychological adjustment of adolescents is not undermined by their ART conception, and that ART families show positive family functioning, with no difference from that enjoyed by adolescents who are spontaneously conceived. These results are not surprising, considering the context in which these children are born: parents pursuing parenthood through ARTs face many hurdles, including infertility, legal and/or financial challenges, and social disapproval, and their children are, by necessity, planned. For sexual-minority-parent families, these burdens add to societal negative attitudes and beliefs that the most appropriate family environment in which to support the welfare of a child is composed of a mother and a father who are heterosexual and fertile.

Despite increasing evidence of the positive functioning of diverse families formed through ARTs, however, research in this field remains highly controversial and emotionally charged. It is therefore imperative that rigorous empirical research continue to inform public dialogue and policy relating to the regulation of access to ARTs for all individuals, regardless of the shape and size their family will assume. While waiting for more inclusive social policies and legal regulations to support families in all their diversity, this article has summarized current knowledge and pointed to what requires further investigation in this area of research.

## Data Availability

Not applicable.
